# EphA3 deficiency in the hypothalamus promotes high-fat diet-induced obesity in mice

**DOI:** 10.7555/JBR.36.20220168

**Published:** 2022-11-28

**Authors:** Jubiao Zhang, Yang Chen, Lihong Yan, Xin Zhang, Xiaoyan Zheng, Junxia Qi, Fen Yang, Juxue Li

**Affiliations:** 1 State Key Laboratory of Reproductive Medicine, Nanjing Medical University, Nanjing, Jiangsu 211166, China; 2 Key Laboratory of Human Functional Genomics of Jiangsu Province, Nanjing Medical University, Nanjing, Jiangsu 211166, China; 3 The Affiliated Eye Hospital of Nanjing Medical University, Nanjing, Jiangsu 210029, China; 4 The Second Affiliated Hospital of Nanjing Medical University, Nanjing, Jiangsu 210011, China

**Keywords:** EphA3, hypothalamus, metabolism, CRISPR-Cas9, obesity

## Abstract

Erythropoietin-producing hepatocellular carcinoma A3 (EphA3) is a member of the largest subfamily of tyrosine kinase receptors—Eph receptors. Previous studies have shown that EphA3 is associated with tissue development. Recently, we have found that the expression of EphA3 is elevated in the hypothalamus of mice with diet-induced obesity (DIO). However, the role of EphA3 in hypothalamic-controlled energy metabolism remains unclear. In the current study, we demonstrated that the deletion of EphA3 in the hypothalamus by CRISPR/Cas9-mediated gene editing promotes obesity in male mice with high-fat diet feeding rather than those with normal chow diet feeding. Moreover, the deletion of hypothalamic EphA3 promotes high-fat DIO by increasing food intake and reducing energy expenditure. Knockdown of EphA3 leads to smaller intracellular vesicles in GT1-7 cells. The current study reveals that hypothalamic EphA3 plays important roles in promoting DIO.

## Introduction

The obesity epidemic has imposed a serious socio-economic burden on the healthcare system worldwide^[1–2]^. The excessive intake of high-fat, high-sugar foods plays a crucial role in the development of obesity by disrupting hypothalamic control of food intake and energy balance^[3]^. The hypothalamus is a pivotal governing center in the regulation of energy homeostasis *via* the ability of several neuronal populations in response to hormone and nutrition-related signals by altering their activity levels^[4–5]^.

In the arcuate nucleus (ARC) of the hypothalamus, there are two functionally distinct neuronal populations, neuropeptide Y (NPY)/agouti-related peptide (AgRP) neurons and pro-opiomelanocortin (POMC) neurons. AgRP neurons are hunger-sensitive neurons, and the activation of these neurons can promote appetite, increase food intake, and reduce energy expenditure^[6–7]^. In contrast, POMC neurons are anorexigenic, and their activation not only reduces food intake but also increases energy expenditure through increasing beige fat activity and inducing white fat browning^[8–9]^. Therefore, to understand the pathogenesis of obesity, it is important to explore the molecular mechanism of hypothalamic neurons in the regulation of energy metabolism. Still, the key genes that drive hypothalamic neuronal dysfunction in obesity remain mostly unidentified.

The erythropoietin-producing hepatocellular carcinoma A3 (EphA3) is a member of EphA receptors that consist of the largest subfamily of tyrosine kinase receptors. Previous studies have shown that EphA3 regulates cell morphology and cell migration during tissue development^[10]^. EphA3 is highly expressed in the axons of the juvenile mouse brain and is involved in the growth of neuronal axons^[11–12]^. The EphA3-knockout mice demonstrated developmental defects of the atrial septum and atrioventricular endocardial cushion, resulting in approximately 75% of mouse deaths within two days after birth^[13]^. Moreover, one study showed that EphA/ephrin regulated insulin secretion and glucose homeostasis *via* mediating islet β-cell communication^[14]^. Recently, we have found that the expression of EphA3 is elevated in the whole hypothalamus of diet-induced obesity (DIO) mice (data not yet published). However, the role of hypothalamic EphA3 in regulating energy balance is unclear.

In the current study, we hypothesized that the deletion of hypothalamic EphA3 might cause disturbances of hypothalamic neuron function, leading to the disruption of energy balance and the development of obesity. By adeno-associated virus (AAV)-mediated CRISPR-Cas9 gene editing technology, we knocked out the expression of EphA3 in the hypothalamus and found that these mice displayed severe obesity phenotype under high-fat diet feeding. The deletion of hypothalamic EphA3 increased weight gain through increased food intake and decreased thermogenesis in diet-induced obese mice. In addition, the knockdown of EphA3 in GT1-7 cells resulted in smaller intracellular vesicles. Together, these results indicate that hypothalamic EphA3 plays important roles in promoting DIO.

## Materials and methods

### Animals

All animals were housed under a 12/12 h light/dark cycle at a temperature of 20 ℃ to 22 ℃. Standard pellet chow and water were provided *ad libitum*. The animal care and procedures were approved by the Animal Core Facility of Nanjing Medical University. NPY-hrGFP (Jax#006417), and POMC-EGFP (Jax#009593) mice were purchased from the Jackson Laboratory (Bar Harbor, ME, USA). The Cre-dependent Rosa26 Cas9 knockin mice were kindly provided by Professor Bin Shen (Nanjing Medical University, China).

### sgRNA design and virus production

For the CRISPR-Cas9 mediated gene editing, the target regions in the genome are the first three exons of the *Epha3* gene in mice with C57BL/6 background. The sgRNAs were designed using the CRISPOR website (http://crispor.tefor.net/) to minimize the off-target effect. The sgRNAs used in this study are listed in ***Table 1***. The sgRNAs were respectively constructed into the AAV plasmid backbone: ITR-U6-sgRNA (backbone)-pCBh-Cre-WPRE-hGHpA-ITR (plasmid #60229, Addgene, Watertown, MA, USA).

**Table 1:** sgRNA sequences targeting the *Epha3* gene

sgRNAs	Sense primers (5′-3′)	Antisense primers (5′-3′)
sgRNA-1	TCAGTTCTCCGGAGCAGCTG	CAGCTGCTCCGGAGAACTGA
sgRNA-2	GCAGCCGAGCAGGACGAGGA	TCCTCGTCCTGCTCGGCTGC
sgRNA-3	CTCTCCATCCTCGTCCTGCT	AGCAGGACGAGGATGGAGAG
sgRNA-4	GAAAACAATTCAAGGAGAGC	GCTCTCCTTGAATTGTTTTC
sgRNA-5	TGGAGCTAAAGTTCACACTG	CAGTGTGAACTTTAGCTCCA

Recombinant AAV9 viral particles were generated by co-transfecting AAV-293T cells of sgRNA plasmids with virus packaging vectors pAAV2/9n (plasmid #112865, Addgene) and pAdDeltaF6 (plasmid #112867, Addgene). Cell transfection was performed using polyethylenimine reagent (Polysciences, Warrington, PA, USA). We changed the cell culture medium 10 h after transfection and harvested the transfected cells and cell culture supernatant after an additional 72-h culture. AAVs were purified and concentrated by iodixanol density-gradient ultracentrifugation. The concentrated AAVs were diluted with PBS to 10^13^ genome copies per mL for* in vivo* experiments. Viral aliquots were stored at −80 ℃ before stereotaxic injection.

Lentiviral particles were generated by cotransfecting 293T cells with virus packaging vectors (pMDL [plasmid #12251, Addgene], pVSVG [plasmid #8454, Addgene], and pRSV-Rev [plasmid #12253, Addgene]). The transfection was performed using polyethylenimine. Six hours after transfection, the medium was changed. Virus supernatant was harvested 24 and 48 h after transfection, 3T3 cells were infected with lentivirus followed by selection in DMEM containing puromycin (2 µg/mL) for two to five days.

### T7 endonuclease Ⅰ assay for genome editing

The T7 endonuclease Ⅰ (T7EI) assay was described previously. Briefly, the genomic sequences flanking the sgRNA target sites were amplified by PCR with Q5 High-Fidelity DNA Polymerase (NEB, Ipswich, MA, USA). The PCR fragments were purified and incubated with T7 endonuclease Ⅰ (NEB) at 37 ℃ for 15 min, followed by gel electrophoresis on a 2% agarose gel. Editing efficiency was calculated using the following formula: Editing efficiency = 100%×[1−(1−% of cleavage product)^1/2^].

### Stereotactic injection of adeno-associated viruses

The application of the Cre-dependent Rosa26 Cas9 knockin mice for the *in vivo* gene editing was described previously^[15]^. The Cas9 mice with C57BL/6 background were used for gene knockout in the hypothalamus. For the stereotactic surgery, eight-week-old Cas9 mice were deeply anesthetized with isoflurane and fixed on a stereotaxic apparatus (RWD Life Science, China) with ear bars. After exposing the skull *via* a small incision, an injection needle was inserted into the brain, and the *Epha3* sgRNA AAVs or control viruses (AAV without sgRNA) was injected at a rate of 25 nL per minute using a micro syringe pump. After injection, the pipette was left in position for another 5 min to allow enough absorption and spreading of AAVs before being withdrawn. The coordinates and injection volume used in the study were as follows: the ARC (anterior-posterior [AP], −1.60 mm; dorsal-ventral [DV], −5.80 mm; left-right [LR], ±0.30 mm, 500 nL/side). The AAV-injected mice were allowed to recover for two weeks and then feed with either chow or high-fat diet. All stereotaxic injection sites in the hypothalamus were verified by sequencing section and imaging. All 'missed' or 'partially injected' animals were excluded from data analysis.

### Body weight and food intake measurements

For virus-transduced knockout experiments, animals were recovered two weeks after surgery to allow sufficient expression of the AAV-expressing transgene. Body weight and food intake were measured weekly.

### Glucose tolerance test

For the glucose tolerance test (GTT), D-glucose (1.25 g/kg, Sigma) was orally administrated to overnight-fasted mice. Blood samples were obtained from the tail vein for glucose measurement immediately before and at the indicated times after injection. Glucose levels were measured using a glucometer (ACCU-CHEK Aviva Plus System, Aviva).

### Metabolic measurement

O_2_ consumption, CO_2_ production, respiratory exchange ratio (RER), locomotor activity, and heat production were monitored using Phenomaster Metabolic Cages (TSE Systems, Germany). Mice were given *ad libitum* access to food and water. Mice were acclimatized in the chamber for at least two days prior to data collection. The measurement was performed at various time points after delivery as indicated in each study. Measurements on O_2_ consumption, CO_2_ production, locomotion, and heat production were collected continuously during the whole measurement period. For all the analyses, data were averaged for 24 h, or for day and night periods.

### Quantitative reverse transcription PCR

Total RNA was extracted by Trizolreagent (Takaza, Japan), and cDNA samples were prepared according to the instructions of PrimeScript RT Reagent Kit (Vazyme, China), and then subjected to quantitative PCR (qPCR) assays using the SYBR Green Master Mix (Vazyme). The reaction system is as follows (20 μL): 10 μL of 2× SYBR Green Master Mix, 2 μL of cDNA template, 0.8 μL of upstream primer, 0.8 μL of downstream primer, and 6.4 μL of sterile water. Reaction conditions: 50 ℃ for 2 min, 1 cycle; 95 ℃ for 10 min, 1 cycle; 95 ℃ for 15 s, 60 ℃ for 1 min, 40 cycles. Three replicate reactions were performed for each sample, and *Gapdh* was used as the internal reference gene to calculate the mRNA expression of the target gene. The primer sequences for qPCR are listed in ***Table 2***.

**Table 2:** Primers used for quantitative reverse transcription PCR

Genes	Sense primers (5′-3′)	Antisense primers (5′-3′)
*Epha3*	ACTATGAAAAGGAGCAAGAGACG	TTCCATATCCCGCTGCTGTC
*Ucp1*	AGGCTTCCAGTACCATTAGGT	CTGAGTGAGGCAAAGCTGATTT
*Dio2*	AATTATGCCTCGGAGAAGACCG	GGCAGTTGCCTAGTGAAAGGT
*Ppargc1a*	TATGGAGTGACATAGAGTGTGCT	CCACTTCAATCCACCCAGAAAG
*Gapdh*	AGGTCGGTGTGAACGGATTTG	TGTAGACCATGTAGTTGAGGTCA

### Western blotting

The total protein from various tissues was extracted using radio-immunoprecipitation assay (RIPA) lysis buffer (Beyotime, Shanghai, China). Protein concentration was assessed by bicinchoninic acid assay. For Western blotting analysis, the extracted protein was separated on 10% polyacrylamide gels and transferred to PVDF membranes. After membranes were blocked with 5% skim milk powder, primary antibodies were incubated overnight at 4 ℃, and secondary antibodies for 1 h at room temperature (RT). The following primary and secondary antibodies with indicated dilutions were used: anti-EphA3 (1:500; ABclonal, China), anti-β-Actin (1:1000; Beyotime). After the membrane was washed three times, the protein bands were exposed with ECL chromogenic solution, and the grayscale analysis was performed with Image J image analysis software (Tanon, Shanghai, China).

### Immunofluorescence staining

After perfusion with 4% paraformaldehyde (PFA), the mouse brains were collected and post-fixed for 12 h in 4% PFA. Then, the brains were dehydrated in a sucrose gradient of 20% to 30% sucrose, embedded in OCT, and sectioned. Tissue sections were blocked with a blocking solution containing normal horse serum, incubated with primary antibodies at 4 ℃ overnight, and fluorophore conjugated secondary antibodies at room temperature for 1 h. The following primary and secondary antibodies with indicated dilutions were used: anti-EphA3 (1:200; ABclonal, China), anti-NeuN (1:200; Millipore, USA), anti-GFAP (1:500; CST, USA); 488-AffiniPure goat anti-mouse IgG (1:500; Thermo Fisher Scientific, USA) and 594-AffiniPure goat anti-rabbit IgG (1:500; Thermo Fisher Scientific). The sections were photographed using the Zeiss LSM800 Laser Scan Confocal Microscope.

### Histological analysis

The tissues were fixed in 4% PFA, dehydrated using gradient alcohol, and then transparently in xylene, paraffin-embedded, and sectioned at a thickness of 5 µm. The sections were stained with hematoxylin and eosin solutions sequentially and then were photographed using the optical microscope (Zeiss, Oberkochen, Germany).

### Morphology examinations

GT1-7 cells transduced with lentivirus were fixed in the buffer containing 2.5% glutaraldehyde at RT for 4 h. The cells were then washed three times in fresh 0.1 mol/L phosphate buffer (pH 7.4) for 10 min each. Subsequently, the cells were fixed in 1% OsO_4_ for 1 h at RT and washed three times in 0.1 mol/L phosphate buffer for 10 min each. Finally, the cells were dehydrated with ethyl alcohol and propylene oxide. The sections were stained with uranyl acetate and then observed using a JEM 1010 transmission electron microscope (JEOL, Japan).

### Statistical analysis

GraphPad Prism 9.0 software was used for statistical analysis, Student's *t*-test was used for comparison between the two groups, and *P*<0.05 was considered statistically significant. The data were expressed as mean±SEM.

## Results

### Characterization of the expression of EphA3 in mouse hypothalamus

**Figure 1:**
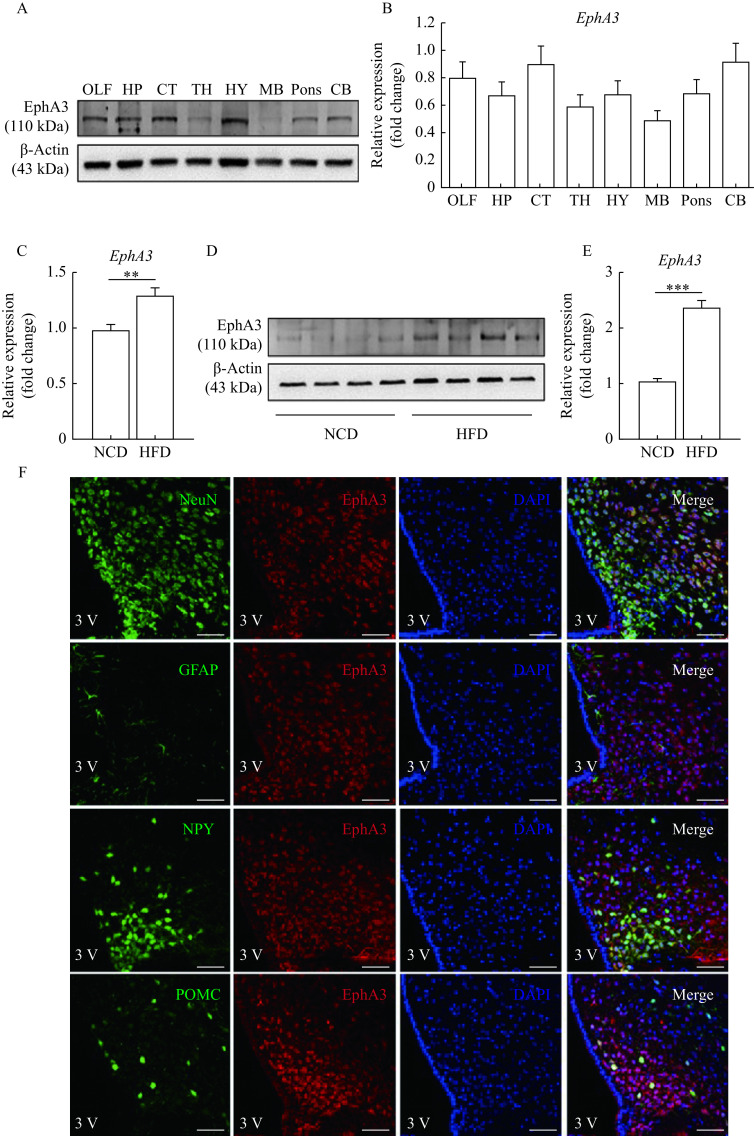
Characterization of EphA3 expression in mouse hypothalamus.

To characterize the expression of EphA3 in the adult mouse brain, we first evaluated protein expression of EphA3 in various mouse brain regions. The result indicated that EphA3 was abundant in the hypothalamus, compared with other regions of the brain (***Fig. 1A*** and ***B***). It is well known that the hypothalamus plays a key role in the energy metabolism. To determine if the hypothalamic EphA3 is related to high-fat DIO, we assessed expression levels of EphA3 in the hypothalamus of high-fat DIO mice. The results showed that the expression of EphA3 in both mRNA and protein levels increased in the hypothalamus of obesity mice (***Fig. 1C***–***E***).

To determine the cell type in which EphA3 was expressed in the hypothalamus, we performed immunofluorescence staining and found that EphA3 was expressed in neurons, but not in glial cells (***Fig. 1F***). Moreover, we found that EphA3 was co-localized with the GFP cells in hypothalamic slices from both NPY-hrGFP transgenic mice and POMC-EGFP transgenic mice, indicating that EphA3 was expressed in AgRP neurons and POMC neurons. Together, these results implicated a potential role of EphA3 in the development of DIO.

### Mutagenesis of *Epha3* gene was achieved by the CRISPR-Cas9 mediated gene editing

**Figure 2:**
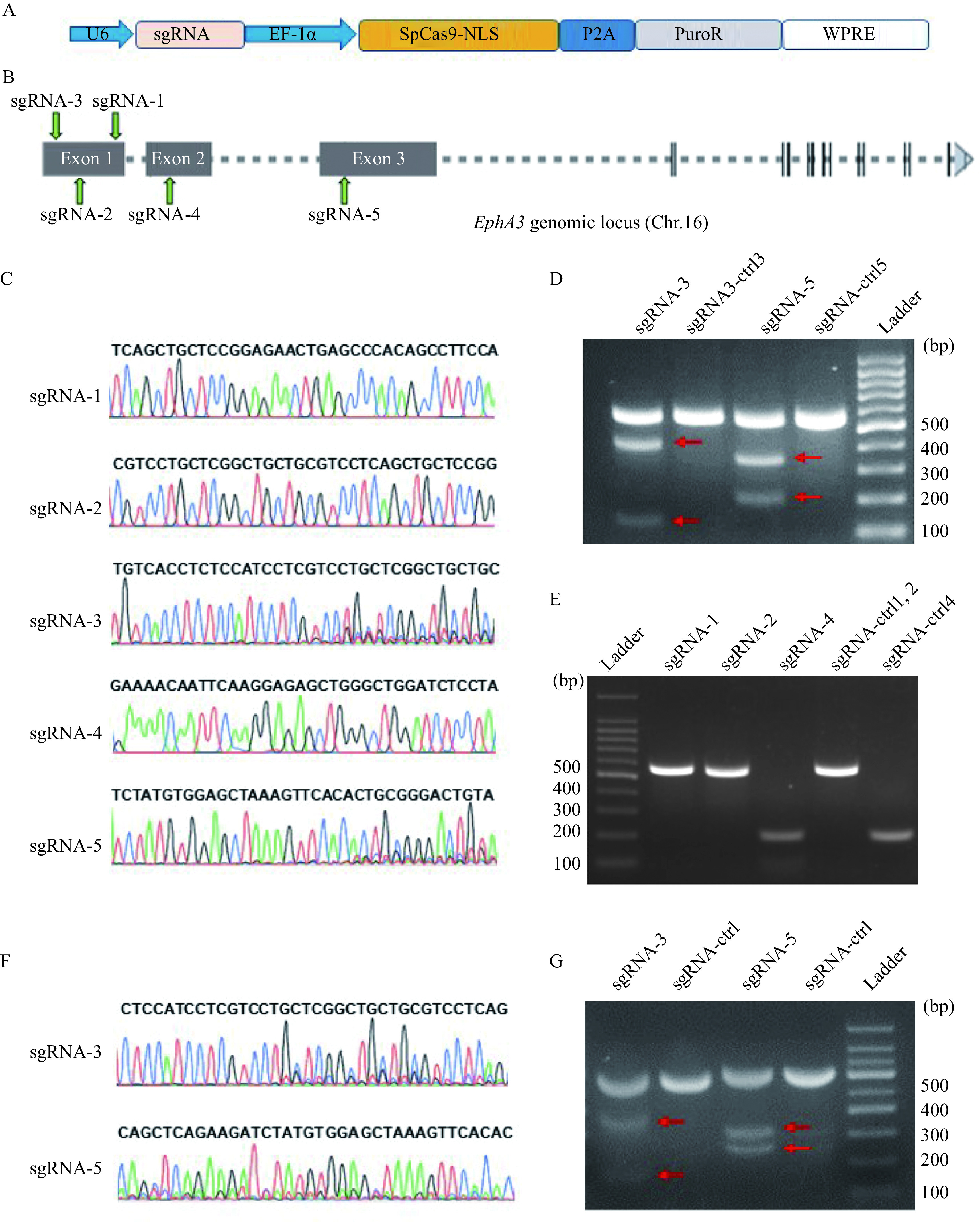
CRISPR/Cas9 mediated target mutagenesis of *Epha3*.

Next, we planned to knock out the *Epha3* gene in mouse hypothalamus to explore the metabolic phenotype. To achieve this purpose, we designed five single guide RNAs (sgRNA) targeting exons 1, 2, or 3 of the mouse *Epha3* gene. We constructed the *Epha3* sgRNAs into lentiviral vectors, produced the lentivirus particles and infected 3T3 cells (***Fig. 2A***). One week after lentivirus infection, DNA sequencing analysis revealed that *de novo* mutations were generated on the *Epha3* gene targeted by sgRNA3 and sgRNA5 (***Fig. 2B***). Consistent with DNA sequencing results, T7EⅠ analysis showed 16.4% and 18.5% mutation rates in 3T3 cells infected with lentivirus containing sgRNA3 or lentivirus containing sgRNA5, respectively (***Fig. 2C–E***). Therefore, we chose sgRNA3 and sgRNA5 for *in vivo* hypothalamus gene editing.

To further confirm the *in vivo* gene editing efficiency of the sRNAs, we injected the AAV2 virus containing sgRNA3 or sgRNA5 into the hypothalamus of ROSA26-LSL-Cas9-P2A-EGFP mice. Consistent with the *in vitro* results, sanger sequencing showed that sgRNA3 and sgRNA5 generated indels three to four bases upstream of the PAM three weeks after injection (***Fig. 2F***). T7EI analysis results showed that the sgRNA3 and sgRNA5 generated similar mutations in target site of the *Epha3* gene (***Fig. 2G***). Thus, these results indicated that CRISPR-Cas9 could efficiently mediate *Epha3* mutagenesis both *in vitro* and *in vivo*.

### Deletion of EphA3 in the hypothalamus promotes DIO in mice

**Figure 3:**
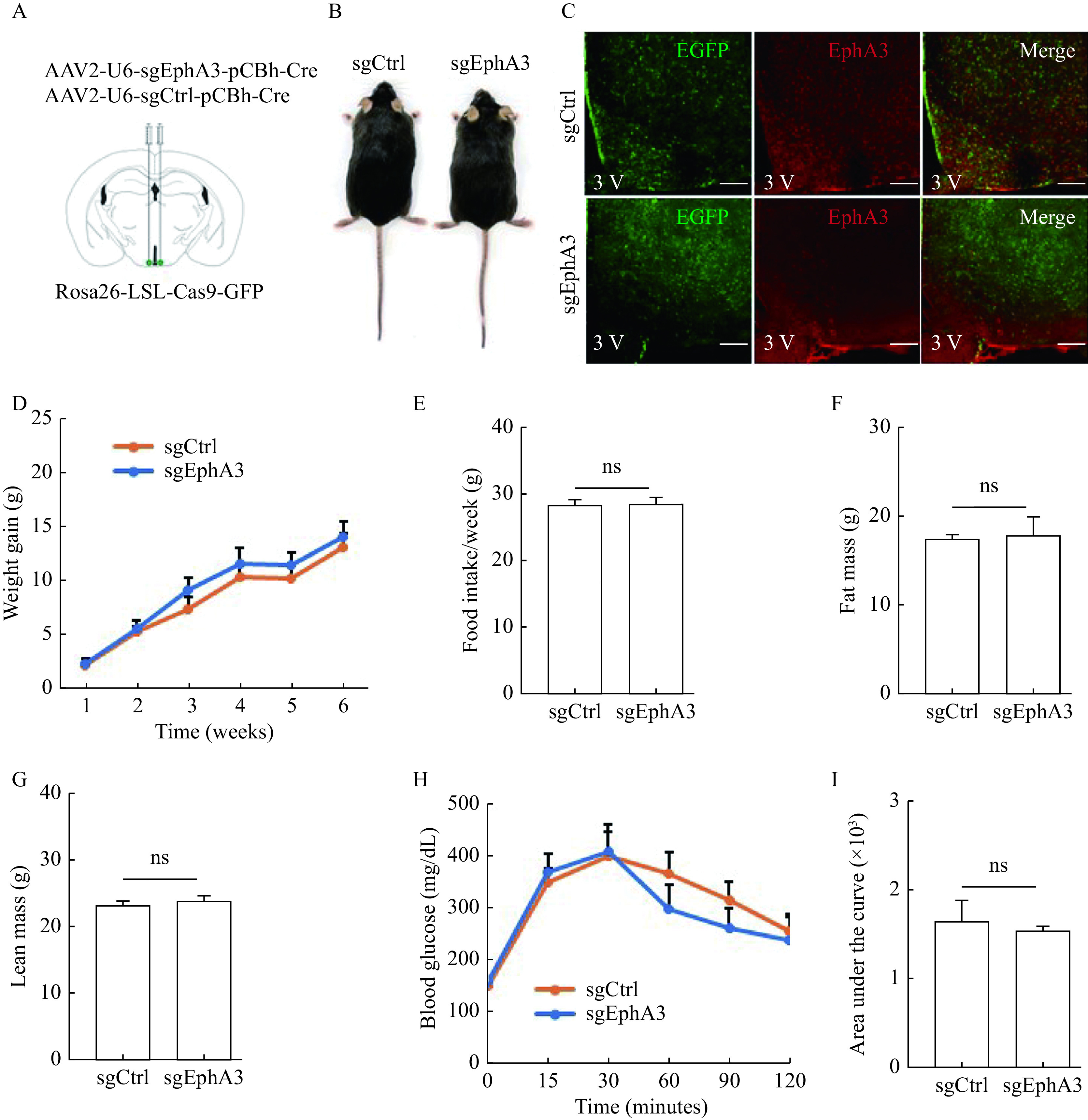
EphA3 knock-out in the hypothalamus does not affect the metabolic phenotype of male mice fed a normal chow diet.

The hypothalamus is known to regulate energy homeostasis and metabolic balance by sensing central and peripheral hormone levels as well as nutrition-related signals. To investigate if EphA3 plays an important role in the energy metabolism in the hypothalamus, we knocked out the *Epha3* gene in the hypothalamus through the CRISPR-Cas9 system (***Fig. 3A*** and ***B***). Immunofluorescence staining results confirmed that the expression of EphA3 was absent in most cells in the hypothalamus injected with AAV-sgEphA3 (***Fig. 3C***).

**Figure 4:**
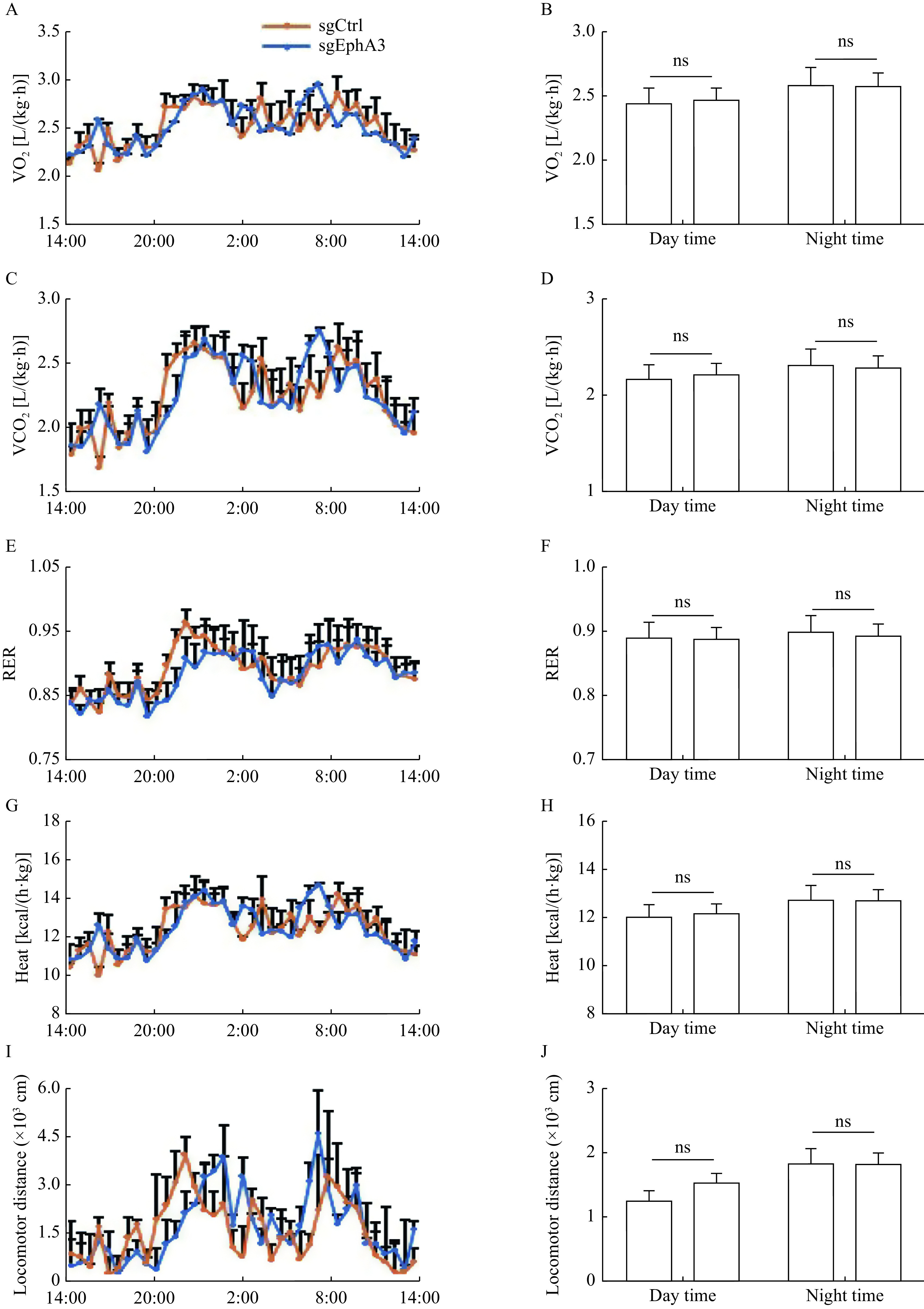
EphA3 deficiency in the hypothalamus does not affect the energy expenditure of male mice fed a normal chow diet.

**Figure 5:**
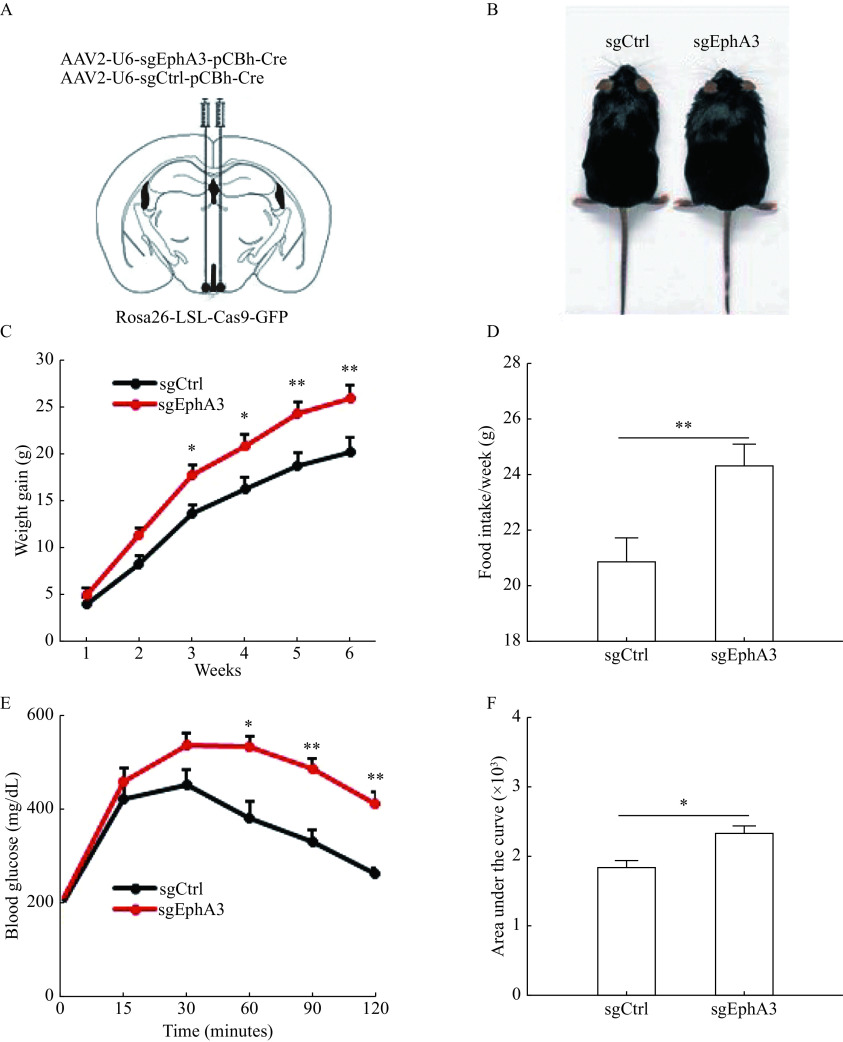
Knock-out EphA3 in the hypothalamus promotes obesity in male mice fed with a high-fat diet.

The AAV-injected mice were recovered two weeks after surgery. Then, the mice were subjected to metabolic phenotype measurements under either normal chow diet feeding or 60% high-fat diet feeding. The results showed that, under normal chow diet feeding, the hypothalamic EphA3 knock-out mice did not show any significant changes in various metabolic phenotype, such as body weight, food intake, body mass composition, blood glucose and energy expenditure (***Fig. 3D–I*** and ***Fig. 4A–J***). However, under the high-fat diet feeding, the hypothalamic EphA3 knock-out mice exhibited a more severe obesity phenotype than control mice. The hypothalamic EphA3 knock-out mice showed significant weight gain and rapid obesity development (***Fig. 5A*** and ***B***). Consistent with body weight results, the hypothalamic EphA3 knock-out mice exhibited significantly higher food intake, and lower tolerance to exogenous glucose (***Fig. 5C–F***). Together, these results indicated that the deletion of EphA3 in the hypothalamus promoted hyperphagia and obesity in the DIO mice.

### Deletion of EphA3 in the hypothalamus affects fat deposition in the DIO mice

**Figure 6:**
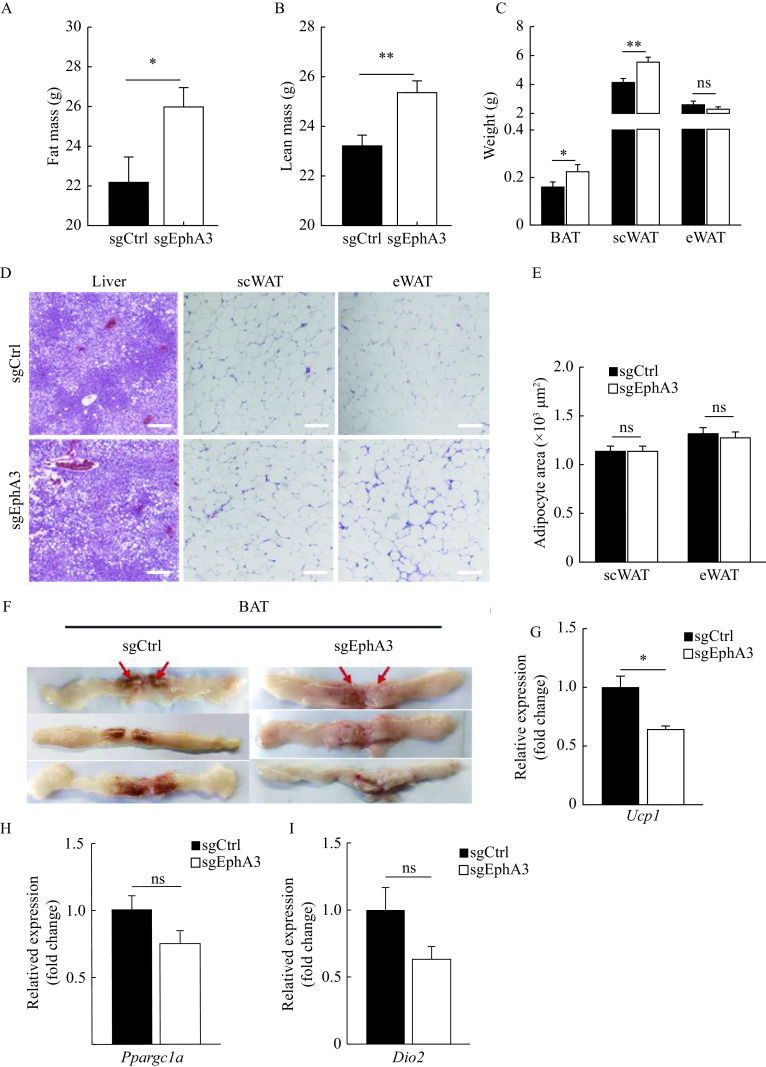
Knock-out of EphA3 in the hypothalamus affects fat deposition in diet-induced obesity mice.

Next, we examined the fat deposition in live and adipose tissue of the hypothalamic EphA3 knock-out mice under the high-fat diet feeding. The weight of brown adipose tissue (BAT) and subcutaneous white adipose tissue (scWAT) were increased in the hypothalamic EphA3 knock-out mice, whereas the weight of epididymal white adipose tissue (eWAT) was not different, compared with that of the control group (***Fig. 6A–C***). H&E staining revealed more severe steatosis and larger lipid droplets in the liver of the hypothalamic EphA3 knock-out mice (***Fig. 6D***). However, H&E staining failed to reveal a significant difference in cell size of scWAT and eWAT between mice treated with AAV-sgEphA3 and control AAV (***Fig. 6E***).

Moreover, we found that the brown adipose tissue of the hypothalamic EphA3 knockout mice showed the morphology of obvious "whitening", compared with that of the control group (***Fig. 6F***). These results implicated that the density of mitochondria in brown adipose cells might reduce in the mice. Next, we measured the expression levels of some genes related to thermogenesis. We found that the mRNA expression level of *Ucp1* in the brown fat of the hypothalamic EphA3 knock-out mice was significantly decreased. The expression levels of *Ppargc1a* and *Dio2* were mildly decreased, without statistical significance (***Fig. 6G–I***). These results above suggested that EphA3 deletion in the hypothalamus might promote obesity through the reduced thermogenesis.

### EphA3 deletion in the hypothalamus reduces energy expenditure in the DIO mice

**Figure 7:**
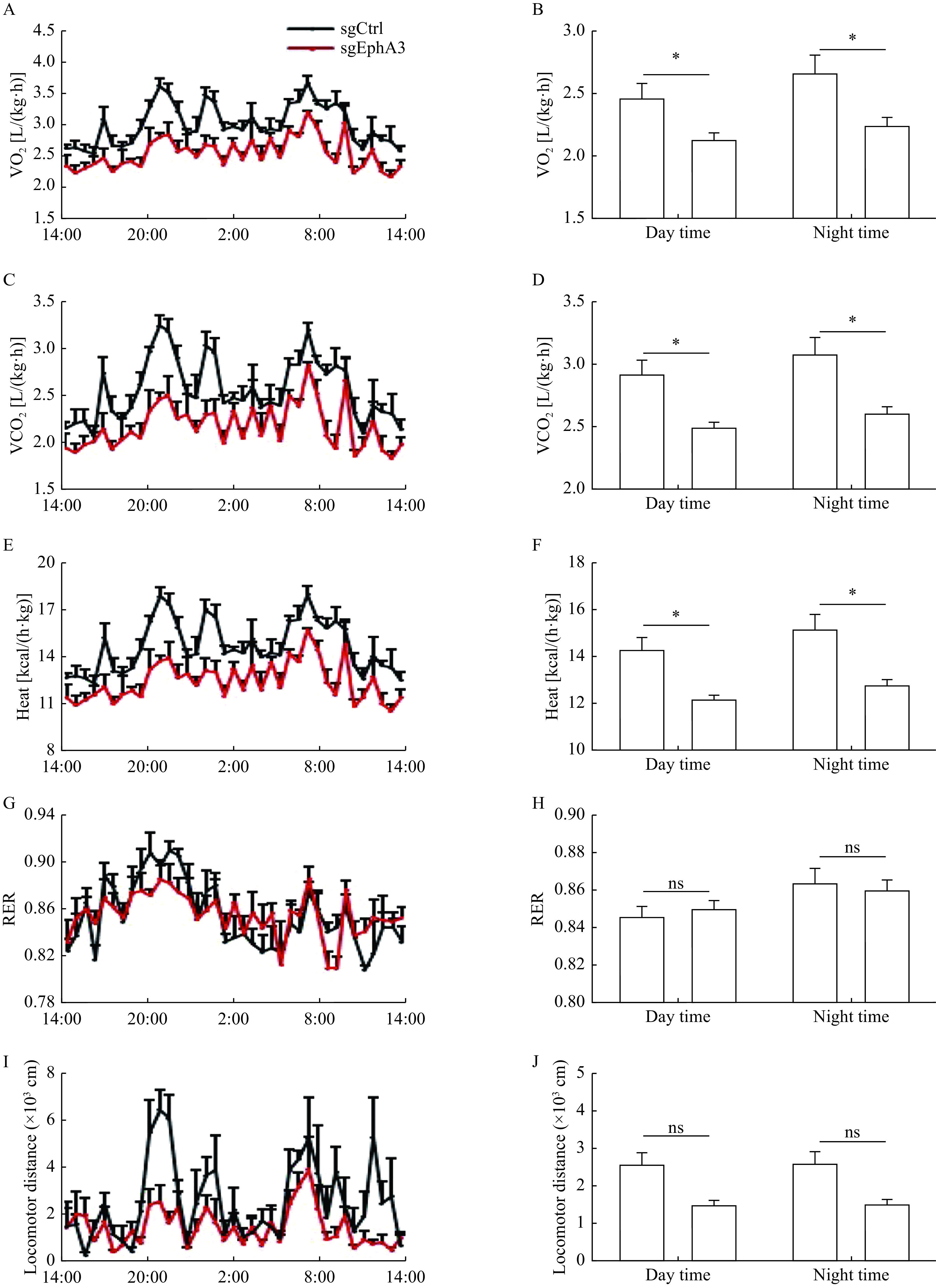
EphA3 deficiency in the hypothalamus reduces energy expenditure in male mice feeding with a high-fat diet.

In addition to food intake, energy expenditure is another key factor determining energy balance. To explore whether energy expenditure contributed to the promotion of DIO in the hypothalamic EphA3 knock-out mice, we measured energy expenditure in male mice two weeks after viral injections. The hypothalamic EphA3 knockout mice exposed to the high-fat diet feeding displayed significant body-weight gain. As expected, we found that hypothalamic EphA3 knockout mice showed a significant decrease in O_2_ consumption, CO_2_ production, and heat production at both light and dark cycles (***Fig. 7A–F***). The respiratory exchange ratio and locomotor activity was not disrupted (***Fig. 7G–J***). Consistent with the reduced UCP1 expression in fat tissues, these results indicated that EphA3 deletion in the hypothalamus promoted DIO by reducing energy expenditure.

### Knockdown of EphA3 results in smaller intracellular vesicles in GT1-7 cells

**Figure 8:**
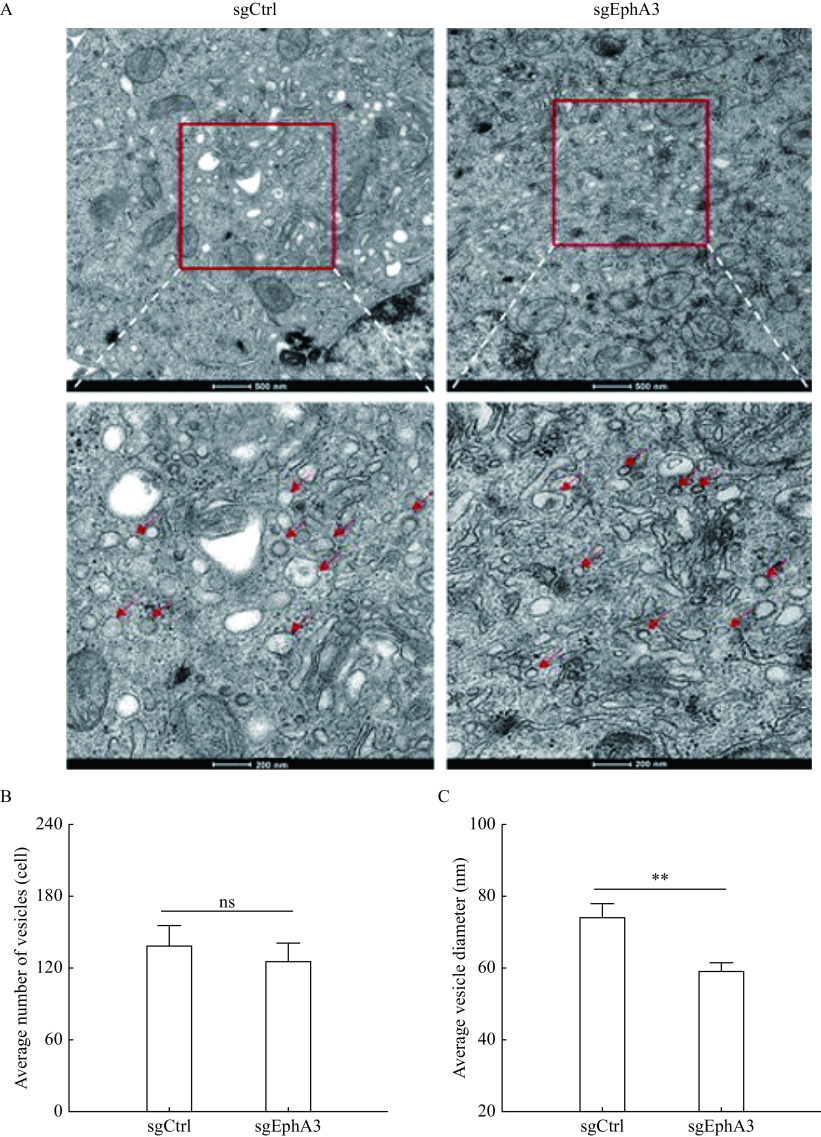
Knock-out of EphA3 results in smaller intracellular vesicles in GT1-7 cells.

GT1-7 cells are immortalized neural cell line isolated from the hypothalamus of transgenic mice and have the typical characteristics of highly differentiated neuroendocrine cells^[16]^. Previous studies have shown that EphA/ephrin mediates islet β-cell communication to regulate insulin secretion^[14]^. To further investigate whether EphA3 plays a similar role in the hypothalamus, we chose GT1-7 cells as an *in vitro* model. We knocked down the EphA3 expression in GT1-7 cells. Electron microscopy results showed that knockdown of EphA3 resulted in smaller intracellular vesicles in GT1-7 cells (***Fig. 8A*** and ***B***), but did not affect the number of vesicles (***Fig. 8C***), suggesting that knockout of EphA3 might affect vesicle formation in the hypothalamus. Together, we speculated that Epha3 deletion may affect hypothalamic neurons that mediates feeding behavior and brown fat activity, leading to a change in body weight.

## Discussion

The hypothalamus plays an important role in maintaining energy homeostasis by integrating nutrient and hormonal signals from peripheral and central sources. In addition, the hypothalamus senses peripheral cold stimuli and activates the BAT activity, which maintains body temperature and leads to non-shivering thermogenesis^[17–18]^. Despite that much progress has been made in understanding the molecular mechanisms by which the hypothalamus regulates energy homeostasis, it is still worthwhile to identify important genes involved in the hypothalamic controlled metabolism. Here, we report that the expression of EphA3 is enriched in the hypothalamus. EphA3 is mainly expressed in neurons including AGRP and POMC neurons. Moreover, its expression level was found to be elevated significantly in the hypothalamus of the DIO mice. These results implied that EphA3 in the hypothalamus might play an important role in DIO.

We performed a physiological study by deleting EphA3 in mouse hypothalamus and measured various metabolic parameters. Our results showed that the hypothalamic EphA3 knock-out mice exhibited a more severe obesity phenotype under the high-fat diet feeding. Specifically, we showed that DIO in the hypothalamic EphA3 knockout mice was promoted through increasing food intake and reducing energy expenditure. Therefore, we speculated that hypothalamic EphA3 might play a protective role in preventing obesity, which may share some similarities with leptin resistance in the hypothalamus of obese mice. Leptin is an anti-obesity cytokine produced by fat cells, and leptin receptors are expressed in various neurons of the hypothalamus. Leptin increases the secretion of the anorexia peptide α-melanocyte stimulating hormone in POMC neurons by binding to hypothalamic leptin receptor, which leads to a reduced energy intake^[19]^. Circulating leptin concentration is significantly increased in obese people, leading to hyperleptinemia^[20–22]^. The knockout of leptin receptor in the CNS neurons leads to severe obesity, hyperinsulinemia, hyperglycemia and hepatic steatosis in male mice^[23]^. Therefore, whether EphA3, as one of the tyrosine kinase receptors, also has a similar resistance mechanism in obesity development remains to be determined.

EphA3 is one member of the largest subfamily of tyrosine kinase receptors—EphA receptors, and it consists of an extracellular ligand binding domain, an intracellular functional domain with tyrosine kinase activity and a transmembrane domain composed of hydrophobic amino acids. Binding of Eph receptors to Ephrin ligands causes Eph/Ephrin to aggregate to form heterotetramers, resulting in intracellular cross-phosphorylation followed by the recruitment of downstream messengers^[24–25]^. In previous studies, investigations related to the EphA3 receptor mainly focused on embryonic development and malignant diseases. EphA3 was demonstrated to play important roles in cancerogenesis^[26–27]^. Moreover, evidence has suggested that EphA3 is involved in fat distribution during adipocyte development^[28]^. However, there is no research report on the role of EphA3 in the hypothalamus controlled energy metabolism. In the current study, we found that hypothalamic EphA3 played an important role in the energy metabolism. We speculate that the functioning of EphA3 in the hypothalamus is mediated by AgRP neurons. AgRP neurons can release Agrp and NPY, promote food intake and reduce energy expenditure^[29–30]^. Optogenetic/chemical genetic manipulation of AgRP^ARC^ → MC4R^dlDRN^ → 5-HT^dmDRN^ neural circuit bidirectionally controls thermogenesis and energy expenditure. A previous study has shown that Epha/ephrin is associatedwith insulin secretion^[14]^. Therefore, we have investigated whether EphA3 plays a similar role in the hypothalamus. AgRP neurons release vesicles containing the neuropeptides NPY and AgRP to regulate the food intake and energy expenditure. We speculate that small vesicles caused by EphA3 deletion may affect the release of neuropeptides NPY and AgRP in AgRP neurons, disrupting its feeding behavior and brown fat activity, and leading to a change in body weight. However, more studies are needed to clarify the detailed mechanism.

In conclusion, we found that the expression of EphA3 was elevated in the hypothalamus of obese mice. The deletion of the hypothalamic *Epha3* gene resulted in an increased feeding and a decreased thermogenesis in male mice fed on a high-fat diet. Our results lay the groundwork for future studies on the mechanism of EphA3 regulation in the energy metabolism.
